# Association between serum liver enzymes and hypertension: a cross-sectional study in Bangladeshi adults

**DOI:** 10.1186/s12872-020-01411-6

**Published:** 2020-03-11

**Authors:** Sadaqur Rahman, Shiful Islam, Tangigul Haque, Rahanuma Raihanu Kathak, Nurshad Ali

**Affiliations:** Department of Biochemistry and Molecular Biology, Shahjalal University of Science and Technology, Sylhet, 3114 Bangladesh

**Keywords:** Liver enzymes, Blood pressure, Prevalence, Hypertension, Bangladeshi adults

## Abstract

**Background:**

Hypertension is a major contributing factor to cardiovascular disease and is a leading cause of death in the world. The association between hepatic enzymes and hypertension has been reported in limited studies and the findings are inconsistent; data from Bangladeshi adults are not available yet. This study was conducted to estimate the prevalence of elevated liver enzymes and evaluate the association of elevated liver enzymes with hypertension in Bangladeshi adults.

**Methods:**

In this cross-sectional study, 302 blood samples were collected from adult participants and analyzed the serum concentrations of alanine and aspartate aminotransferase (ALT, AST), alkaline phosphatase (ALP), and γ-glutamyltransferase (GGT) and other markers related to hypertension. Hypertension was defined as resting SBP ≥ 140 mmHg and/or DBP ≥ 90 mmHg. Associations between elevated liver enzymes and hypertension were evaluated by multinomial logistic regression.

**Results:**

The mean concentrations of serum ALT, AST and GGT were significantly higher in the hypertensive group compared to the normotensive group (*p* < 0.01, *p* < 0.01 and *p* < 0.001, respectively). Overall, 49.2% of subjects in the hypertensive group and 38.1% of individuals in the normotensive group had at least one or more elevated liver enzymes. The prevalence of elevated ALT, AST, and GGT was significantly higher among participants in the hypertensive group compared to the normotensive group (*p* < 0.01, *p* < 0.01 and *p* < 0.001, respectively). An increasing trend for elevated liver enzymes was observed with increasing blood pressure. Serum ALT and GGT showed an independent relationship with hypertension.

**Conclusions:**

The prevalence of elevated liver enzymes was higher in hypertensive individuals. Increased serum ALT and GGT activities were positively associated with hypertension in Bangladeshi adults.

## Background

Hypertension is a major contributing factor for cardiovascular disease (CVD) and one of the leading causes of death in the world [1, 2]. The world prevalence of hypertension was 26% in 2000 which is projected to be increased by 29.2% in 2025 [3]. The incidence of hypertension is increasing in developing countries along with developed countries [4]. In the Asian region, hypertension has become a significant concern affecting over 35% of the adults and the countries especially in South-East Asia are particularly facing the increasing burden of hypertension [5, 6]. The incidence of hypertension in Bangladesh has increased rapidly in the past years [7, 8]. Hypertension and its complications are responsible for a significant portion of the death of the Bangladeshi population and a burden for the national economy. Early diagnosis and management of increased blood pressure before hypertension development may be cost-benefit in terms of reducing premature morbidity and mortality in general people [9].

The interrelationship between liver dysfunction and the development of hypertension is being increasingly recognized. The liver is a vital organ in metabolism that plays numerous roles included synthesis, degradation, storage, and biotransformation of bio-molecules in the human body [10]. The liver enzymes alanine and aspartate aminotransferase (ALT and AST), γ-glutamyltransferase (GGT), and alkaline phosphatase (ALP) have been widely used as a good marker of liver health [11]. The elevated levels of ALT, AST, and GGT reflect an excess fat deposition in the liver, a condition termed as nonalcoholic fatty liver disease (NAFLD). These enzymes are suggested to have substantial clinical and epidemiological significance as convenient surrogate markers of NAFLD and related liver dysfunction [12, 13]. Some epidemiological studies have demonstrated an association of ALT and GGT with metabolic syndrome, CVD and type 2 diabetes [14–17]. In previous studies, CVD has been demonstrated as a leading cause of death in NAFLD, with higher rates coinciding with increased liver-related mortality throughout follow-up investigations [18–20].

An association between higher serum GGT levels and hypertension has been reported in some longitudinal and cross-sectional studies [15, 21–24]. However, most of the previous studies assessed the relationship that included only one or two hepatic enzymes and their findings were inconsistent. The epidemiological data concerning the extent of elevated liver enzymes in the Bangladeshi hypertensive individuals are not available yet. To address these issues, we conducted a cross-sectional study to examine the association of all four liver enzymes with hypertension in Bangladeshi adults.

## Methods

### Study participants and area

This cross-sectional study was conducted between October 2017 and September 2018 at the Department of Biochemistry and Molecular Biology, Shahjalal University of Science and Technology, Sylhet, Bangladesh. More than 350 participants aged above 18 years were invited randomly to take part in the study, among them 302 subjects agreed to participate. The participants included general adults from Sylhet city regions, academic and non-academic staffs and university adult students. The inclusion criteria were: both gender, aged above 18 years, free from severe chronic illness and willing to participate. Exclusion criteria: participants with a history of hepatotoxic drugs intake, alcohol intake (alcohol consumption is generally prohibited in Bangladesh from religion restriction), and severe chronic or acute evidence of liver diseases reported by participants were excluded from the study. Written informed consent obtained from all participants before inclusion in the study. This study was approved by the internal Ethics Committee at the Department of Biochemistry and Molecular Biology of Shahjalal University of Science and Technology. All steps in the method section were conducted out following the relevant guidelines and regulations.

### Anthropometric and blood pressure data

Trained personnel measured the anthropometric and blood pressure (BP) data and recorded in the questionnaire form according to the standard procedure described elsewhere [25–27]. An individual’s body weight and height were measured to calculate the BMI (kg/m^2^). Using a digital BP machine (Omron M10, Omron Corporation, Tokyo, Japan), individuals BP was measured on the left arm in a sitting position after the participant rested for 10 min. The first BP measurement was discarded to avoid possible effects of anxiety, and the mean value of the second and third measurements was count for systolic blood pressure (SBP) and diastolic blood pressure (DBP). The participants were requested to avoid coffee, tea and smoking for 30 min before BP measurements. Physical activity was categorized as low, medium and adequate based on participation in any activities such as jogging, bicycling, swimming or daily sports. The questionnaire also asked about the smoking status of the participants (yes or no). Individual food habits and brief lifestyle information were also recorded in the questionnaire form.

### Sample collection and biochemical analysis

The participants were at least 10–12 h of overnight fast before providing the blood samples. From each participant, about 5 ml of the venous blood was drawn in a plain dry vacutainer tube using disposable syringes. Serum was separated and stored at − 80 °C in the laboratory until biomarkers analysis. Serum concentrations of glucose, total cholesterol (TC), triglycerides (TG), albumin and total protein were measured by colorimetric methods. Activities of serum liver enzymes ALT, AST, GGT and ALP were measured by kinetic methods. All measurements were done using commercially available diagnostic kits (Human Diagnostic, Germany, except GGT from Vitro Scient, Egypt) with a biochemistry analyzer (Humalyzer 3000, USA).

### Diagnostic criteria

The liver enzymes at elevated levels were defined as one or more measurement of: AST > 35 U/L in men/ > 31 U/L in women, ALT > 45 U/L in men/ > 34 U/L in women,, GGT > 55 U/L in men/ > 38 U/L in women [28] and ALP > 128 U/L in men/ > 98 U/L in women [29]. Hypertension (stage 2) was defined as resting SBP ≥ 140 mmHg and/or DBP ≥ 90 mmHg or by treatment for hypertension [30, 31].

### Statistical data analysis

Baseline data are expressed as mean ± standard deviation, whereas, the categorical data are mentioned as percentages. Pearson’s correlation coefficient test (two-tailed) was performed to examine the correlation between hepatic markers and baseline variables. The differences between anthropometric and baseline characteristics in the gender and case-control groups were done by independent sample t-test. Associations between liver enzymes and hypertension were evaluated by multinomial logistic regression analysis. A *p*-value of < 0.05 was set statistically significant. Statistical data analyses were done using IBM SPSS software (version 23).

## Results

### Baseline characteristics of the study cohort

**Fig. 1:**
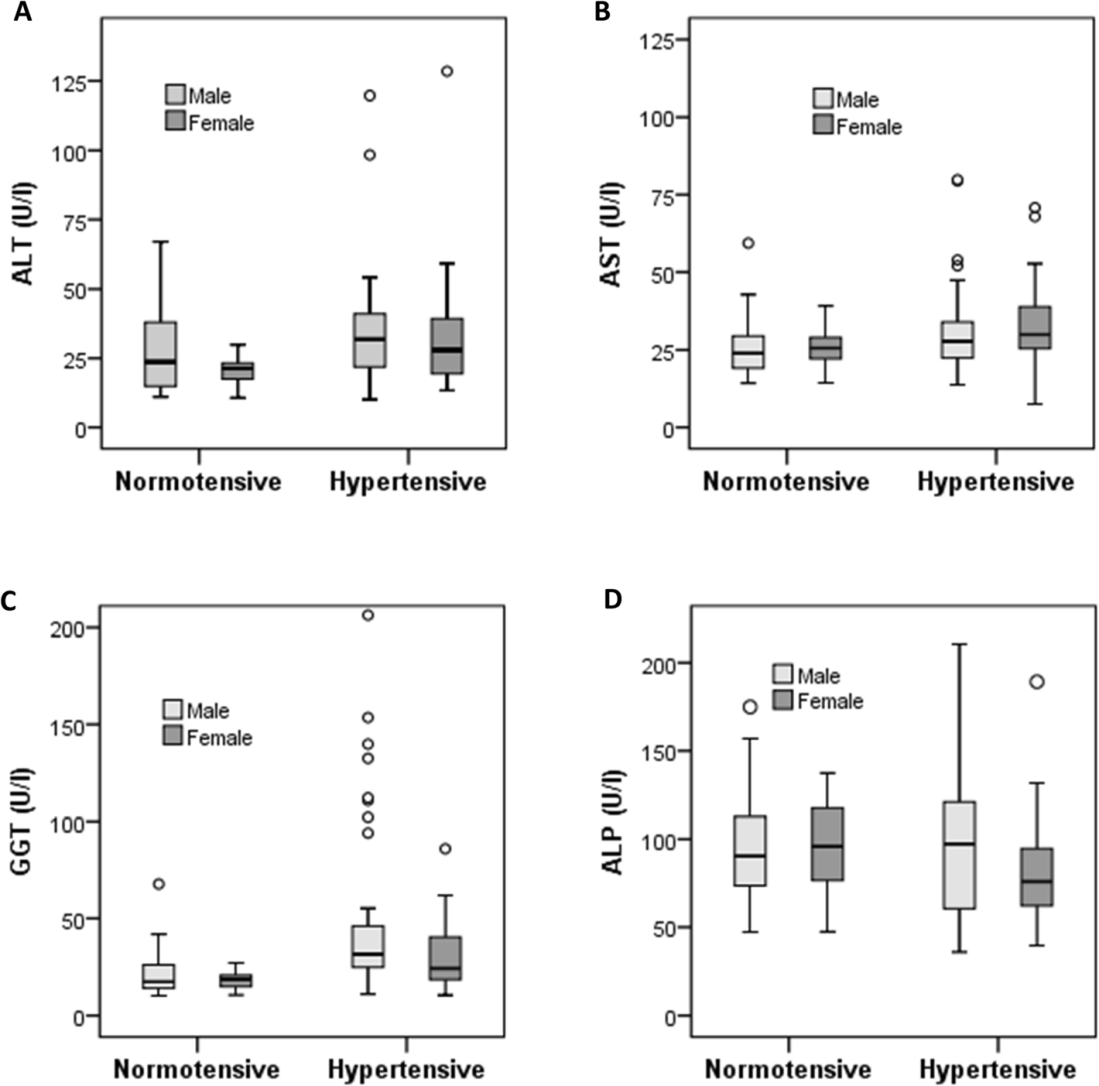
Levels of ALT (**a**), AST (**b**), GGT (**c**) and ALP (**d**) in normotensive and hypertensive group by gender. The scale in the Y-axis is not similar for all liver enzymes. The box represents the central data of distribution where upper and lower limits of the box indicate 25th and 75th percentiles (first quartile or Q1 and third quartile or Q3, respectively) and the median value is presented as a line inside the box. In the box plot outlier values are more than 1.5 times the interquartile range (IQR) and the Q1 and Q3, respectively”

**Fig. 2:**
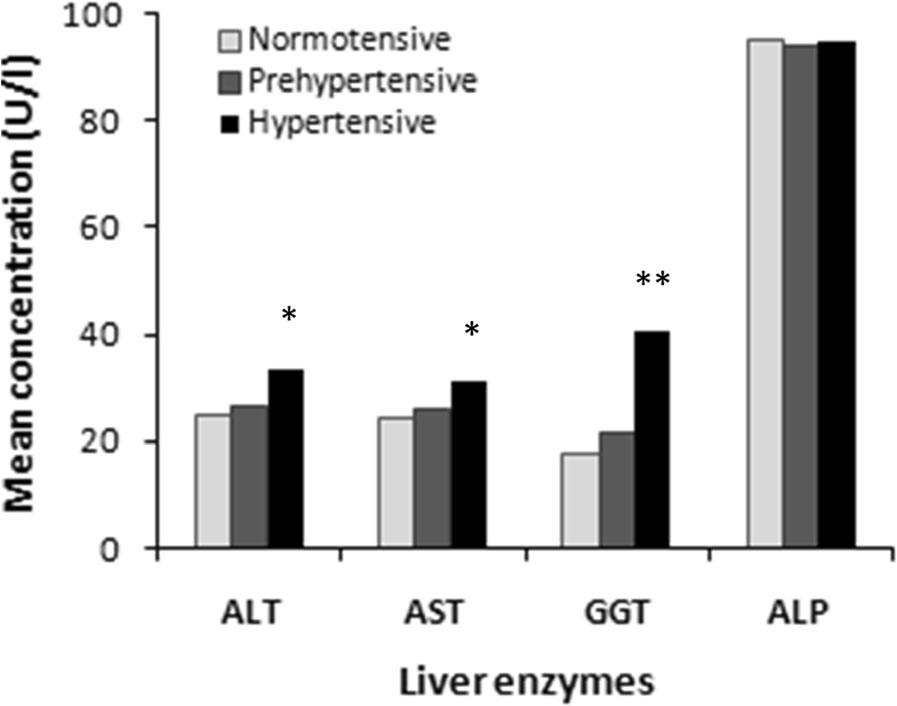
Levels liver enzymes in normotensive, prehypertensive and hypertensive group. ^*^P < 0.01 and ^**^P < 0.001 when compared to normotensive group

The baseline characteristics of the study subjects are presented in Table 1. Out of 302, 198 were normotensive (152 male and 46 female) and 104 were hypertensive individuals (74 male and 30 female). The average age for normotensive and hypertensive subjects was 37.2 ± 14.6 and 44.0 ± 15.2 years, respectively. The participants in the hypertensive group had a significantly higher mean BMI (25.7 ± 3.7 kg/m^2^) than the participants in the normotensive (23.3 ± 3.5 kg/m^2^) group (*p* < 0.001). The mean concentrations of ALT, AST, GGT were significantly higher in the hypertensive group compared to the normotensive group (*p* < 0.01, *p* < 0.01 and *p* < 0.001, respectively). A variation has been observed for liver enzymes among male-females in both normotensive and hypertensive groups (Fig. 1). Male participants in the normotensive group had higher mean concentrations of ALT and GGT whereas ALP concentration was higher in females. In the hypertensive group, mean GGT and ALP concentrations were higher in males and AST was higher in females but no differences were observed for ALT concentration between male and female participants. An increasing trend of liver enzymes concentrations was observed in the BP groups (Fig. 2). The mean levels of serum TC, TG and glucose were also significantly higher in the hypertensive group (*p* < 0.001). In both groups, no significant differences were found for serum albumin, total protein, smoking status, physical activity and raw salt intake.

**Table 1:** Baseline characteristics of the hypertensive and normotensive participants

	Normotensive	Hypertensive	*p*-value
*N*	198	104	–
Male/female	152/46	74/30	–
Age (yrs)	37.2 ± 14.6	44.0 ± 15.2	0.000
BMI (kg/m^2^)	23.3 ± 3.5	25.7 ± 3.7	0.000
SBP (mm Hg)	121.2 ± 9.5	145.7 ± 16.5	0.000
DBP (mm Hg)	77.1 ± 6.2	91.4 ± 7.4	0.000
ALT (U/l)	26.0 ± 13.7	33.7 ± 19.5	0.006
AST (U/l)	25.7 ± 8.3	31.2 ± 13.5	0.003
GGT (U/l)	20.4 ± 9.5	40.8 ± 33.5	0.000
ALP (U/l)	96.0 ± 29.6	94.8 ± 36.9	0.830
Glucose (mg/dl)	102.8 ± 28.7	109.2 ± 24.2	0.323
TG (mg/dl)	128.4 ± 60.0	221.1 ± 133.7	0.000
TC (mg/dl)	188.7 ± 69.6	259.3 ± 100.6	0.000
Serum albumin (mg/dl)	48.9 ± 10.8	50.7 ± 19.9	0.489
Total Protein (mg/dl)	78.6 ± 28.0	80.6 ± 27.2	0.650
Smoking status (%)			0.328
Yes	22.9	16.5	
No	77.1	83.5	
Physical activity (%)			0.476
High	7.1	5.1	
Moderate	67.1	77.2	
Low	25.7	17.7	

Data are presented as mean ± SD. *P*-values are obtained from Independent sample t-test

### Prevalence of elevated liver enzymes

The prevalence of elevated liver enzymes in both groups are presented in Table 2. Overall, 49.2% of participants in hypertensive and 38.1% participant’s in the normotensive group had at least one or more elevated liver enzymes. The prevalence rate was significantly higher in hypertensive group (ALT 20.6% vs 13.8%, *p* < 0.01; AST 27.5% vs 17.7%, *p* < 0.01; GGT 19.6% vs 2.3%, *p* < 0.001 and ALP 20.6% vs 16.9%, *p* > 0.05) compared to the normotensive group (Table 2). In the hypertensive group, the most common liver enzymes abnormalities were found in female participants, whereas, in the normotensive group, a variation was observed between male-female groups.

**Table 2:** Prevalence of elevated liver enzymes in normotensive and hypertensive group by gender

		Normotensive (*n* = 198)			Hypertensive (*n* = 104)		
		Male	Female	Total	Male	Female	Total
ALT ^a^	Elevated (%)	17.0	3.3	13.8	16.2	32.1	20.6
	Normal (%)	83.0	96.7	86.2	83.8	67.9	79.4
AST ^b^	Elevated (%)	17.0	20.0	17.7	23.0	39.3	27.5
	Normal (%)	83.0	80.0	82.3	77	60.7	72.5
GGT ^c^	Elevated (%)	2.0	3.3	2.3	14.9	32.1	19.6
	Normal (%)	98.0	96.7	97.7	85.1	67.9	80.4
ALP ^d^	Elevated (%)	9.0	43.3	16.9	20.3	21.4	20.6
	Normal (%)	91.0	56.7	83.1	79.7	78.6	79.4

^a^*p* < 0.01, ^b^*p* < 0.01, ^c^*p* < 0.001, ^d^ > 0.05, when compared total prevalence of elevated liver enzymes between normotensive and hypertensive groups

### Correlation between liver enzymes and hypertensive risk factors

The correlations of liver enzymes with common risk factors of hypertension development are summarized in Table 3. AST and GGT showed a significant association with the age of the participants (*p* < 0.05 and *p* < 0.001, respectively). All liver enzymes except ALP showed a significant association with both SBP and DBP. ALP and GGT showed a significant positive association with glucose concentration. ALT, AST and GGT were significantly correlated with TG, where AST and GGT were correlated with TC. The magnitude of these correlations was stronger for AST and GGT. All liver enzymes showed a negative association with serum albumin, whereas, ALT and GGT showed a significant positive association with serum total protein.

**Table 3:** Correlation between liver enzymes and baseline characteristics of the participants

	ALT		AST		ALP		GGT	
	Correlation (*r*)	*p*-value	Correlation (*r*)	*p*-value	Correlation (*r*)	*p*-value	Correlation (*r*)	*p*-value
Age	0.073	0.482	0.158	0.049	0.108	0.189	0.298	0.015
BMI	0.149	0.064	0.189	0.019	0.078	0.459	0.132	0.104
SBP	0.157	0.049	0.161	0.045	0.058	0.624	0.192	0.018
DBP	0.222	0.005	0.222	0.005	0.049	0.642	0.246	0.002
Glucose	0.068	0.517	0.063	0.433	0.177	0.029	0.257	0.001
TG	0.300	0.000	0.381	0.000	0.081	0.350	0.530	0.000
TC	0.113	0.186	0.281	0.001	0.056	0.617	0.225	0.009
Albumin	−0.035	0.663	−0.157	0.052	−0.041	0.621	−0.095	0.244
Total protein	0.258	0.002	0.143	0.083	0.110	0.197	0.236	0.004

Correlation was analyzed using Pearson’s Correlation Coefficient test (two-tailed)

### Association of liver enzymes with hypertension

Table 4 shows the risk of hypertension increased along with the higher concentrations of ALT and GGT, which remained significant even in a multivariate-adjusted logistic model. In model 1, age and sex were adjusted and ALT and GGT showed significant association with hypertension. In model 2, BMI, fasting blood glucose, TG and TC were adjusted, in model 3, further serum albumin and total protein were adjusted and in model 4, additionally smoking status and physical activity were adjusted. In all models, ALT and GGT showed a significant positive association with hypertension (*p* < 0.01 for model 1 and *p* < 0.05 for model 2–4).

**Table 4:** Association of liver enzymes with hypertension

	ALT		AST		GGT		ALP	
	OR (95% Cl)	*p* -value	OR (95% Cl)	*p* -value	OR (95% Cl)	*p* -value	OR (95% Cl)	*p* -value
Model 1	1.04 (1.01–1.07)	0.007	1.04 (0.99–1.08)	0.096	1.07 (1.02–1.12)	0.009	0.99 (0.98–1.00)	0.279
Model 2	1.04 (1.00–1.07)	0.023	1.03 (0.97–1.08)	0.336	1.07 (1.00–1.13)	0.024	0.98 (0.97–1.01)	0.209
Model 3	1.05 (1.00–1.09)	0.019	1.05 (0.99–1.12)	0.119	1.08 (1.01–1.16)	0.023	0.99 (0.97–1.00)	0.209
Model 4	1.06 (1.01–1.11)	0.014	1.08 (1.00–1.16)	0.038	1.09 (1.01–1.18)	0.028	0.99 (0.97–1.01)	0.409

Model 1: adjusted for age and sex

Model 2: Model 1 plus, BMI, glucose, TG and TC

Model 3: Model 2 plus albumin and total protein

Model 4: Model 3 plus smoking status and physical activity

## Discussion

The present study demonstrates that serum ALT and GGT are positively associated with the prevalence of hypertension. This study provides the first information on the association between liver enzymes and hypertension among Bangladeshi adults.

In the present study, the prevalence of elevated hepatic enzymes (ALT, AST, and GGT) was significantly higher among hypertensive individuals. The serum level of ALT was about one and a half and GGT was two times higher in hypertensive subjects than in the normotensive individuals. When age was taken into account, elevated GGT levels were more likely to be found in persons at high risk of hypertension. A high prevalence of elevated levels of ALT and GGT demonstrated a higher risk for hypertensive females and males than their normotensive counterparts. A similar result was found in a previous study that reported a high prevalence of elevated ALT in the hypertensive group compared to the normotensive group [9].

In our study, both SBP and DBP were significantly associated with ALT, AST and GGT. However, in regression analysis, only ALT and GGT were significantly associated with hypertension even after adjustment of potential confounders. Our results for GGT are in agreement with the findings of previous prospective studies [15, 21–24, 32] that showed baseline serum GGT was an independent risk factor for hypertension development. We also observed an increasing trend for the mean levels of ALT, AST and GGT in the prehypertension and hypertensive group compared to the normotensive group. Higher levels of GGT were previously reported in prehypertensive Korean, Japanese, Chinese, and US adults [33–36].

In the present study, serum ALT showed an independent association with hypertension in Bangladeshi adults. A similar finding was observed in a previous study that reported ALT as a potential indicator of hypertension in Chinese senior adults [9]. There is no simple explanation for why a serum ALT showed an independent association with hypertension in the Bangladeshi population. One possibility may be that hypertensive individuals develop NAFLD after a long period of elevated blood pressure [37]. The postulated mechanism could be that increased blood pressure activates pro-inflammatory responses such as TNF-α and interleukin adiponectin and leptin that contribute to hepatotoxicity [38]. This needs further investigation. Serum AST and ALP did not show a significant association with hypertension in the present investigation. Up to now, a very limited number of studies evaluated the association of AST and ALP with hypertension and their findings are inconsistent [39, 40]. Moreover, a wide variation has been observed on the prevalence of elevated liver enzymes in the previous studies. Different reference values, age range, ethnicity, and demography might be considerable factors for the observed variations of these studies.

The biological mechanism underlying the relationships between hepatic enzymes and hypertension remains unclear. Study evidence suggests a link of NAFLD with CVD [21]. Some cross-sectional studies showed a higher incidence of NAFLD in hypertensive individuals, as compared with those with normal BP [37, 41]. A recent follow-up study showed that fatty liver development is related to hypertension in Korean adults [42]. On the other hand, a potential mechanism for the link between GGT and hypertension might be related to oxidative stress and the role of cellular GGT in the catabolism of extracellular antioxidant glutathione [21, 35]. Also, it has been reported that cellular GGT may be related to reactive oxygen species production in the presence of transition metals [43]. In parallel, oxidative stress is documented to be associated with hypertension [44] and antioxidant enzyme genes polymorphisms, including few of the glutathione-S-transferase genes, have been reported to be correlated with the risk of hypertension in the general adults [45, 46]. Further investigations are required to confirm the postulated mechanisms between elevated liver enzymes and incident hypertension in the general population.

The strength of the present study included an adjustment for well-known hypertensive risk factors including age, BMI, lipids, smoking and physical activities to examine the relationships. However, some limitations of the present study should be considered. First, we measured the hepatic enzymes at baseline level which may not represent the long-term profile. The second limitation of the present study is a small sample size. Third, the cross-sectional nature of the study may preclude any casual relationship between hepatic enzymes and hypertension. Moreover, hepatitis B and C infection were not measured among the participants that may have effects on elevated hepatic enzymes.

## Conclusion

A high prevalence of elevated liver enzymes was found in hypertensive individuals and this elevation was higher in females than in the males. An increasing trend of elevated liver enzymes was found in the prehypertensive and hypertensive group compared to the normotensive group. Serum ALT and GGT activities were positively associated with the prevalence of hypertension in Bangladeshi adults. Results of this study suggest that monitoring of ALT, GGT levels could help in the diagnosis of hypertension. Prospective large scale studies are needed to investigate the underlying mechanisms between liver enzymes and the incidence of hypertension in the general population.

## Data Availability

The datasets used and analyzed during the present study are available from the corresponding author on reasonable request.

## Abbreviations

BMI: Body mass index
TC: Total cholesterol
TG: Triglycerides
